# Agile Leadership and Perceived Career Success: The Mediating Role of Job Embeddedness

**DOI:** 10.3390/ijerph19084834

**Published:** 2022-04-15

**Authors:** Bulent Akkaya, Mirela Panait, Simona Andreea Apostu, Yesim Kaya

**Affiliations:** 1 Ahmetli Vocational High School, Manisa Celal Bayar University, 45450 Manisa, Turkey; bulent.akkaya@cbu.edu.tr; 2 Department of Cybernetics, Economic Informatics, Finance and Accounting, Petroleum-Gas University of Ploiești, 100680 Ploiești, Romania; 3 Institute of National Economy, 050711 Bucharest, Romania; simona.apostu@csie.ase.ro; 4 Department of Statistics and Econometrics, Faculty of Economic Cybernetics, Statistics and Informatics, Bucharest University of Economic Studies, 010374 Bucharest, Romania; 5 Faculty of Economics, Administrative and Social Sciences, Istanbul Gelisim University, 34310 Istanbul, Turkey; yesimkocyigit@gmail.com

**Keywords:** manager, agile leadership, career success, job embeddedness, structural equation modelling, VUCA

## Abstract

Agile leadership is an important managerial function in which responsiveness and innovation appear to be essential elements for the long-term development and success of any business. The world has become increasingly volatile, uncertain, complex, and ambiguous (VUCA) during and post COVID-19. Managers are required to possess agile leadership to facilitate their employees’ successful careers. Therefore, this study aims to find out the relationship between agile leadership and career success by examining the mediation of job embeddedness in healthcare organizations. The descriptive research design and survey method were employed in this study. The data were collected by using three scales from healthcare employees in healthcare organizations in Turkey. Hypotheses were tested using structural equation modelling (SEM). The data were analysed by using SPSS and AMOS programs. The findings of this study showed that agile leadership behaviours enhance career success. Moreover, the relationship between agile leadership and career success is mediated by job embeddedness. The role of agile leadership in promoting employees’ career success has rarely been studied in the literature. This is one of the first studies to examine the effect of agile leadership on career success along with the mediating role of job embeddedness. Healthcare managers have faced many critical challenges at their workplace during the COVID-19 pandemic. Through the lens of managing efficient healthcare organizations in many contexts, this research sheds some important light on the association between agile leadership, career success, and job embeddedness. Managers with high agility levels used strategies such as group decision making, problem solving, effective internal and external communication, and adaptation to uncertain environment in order to increase their career success.

## 1. Introduction

Leaders have an essential influence on their employees’ perceived career success and their roles are considered most effective in assuring professional success in today’s dynamic work environment. The research into employee career success has attracted the interest of the academic world, as an awareness of the antecedents of employee career success is critical to building qualified employees and attaining organizational success [1,2]. There has been an increase in research studies on the factors that influence career success in a number of occupations, including healthcare [3,4].

To decrease healthcare employees’ turnover, it is critical to investigate the factors that might contribute to their career success [5]. In this context, this study will explore the relationship between agile leadership and perceived career success. Furthermore, the factors which influence employees’ career success were also investigated. Employees should aim for career success since it enhances professional, on-the-job behaviours necessary for improving work quality and performance [6,7]. Career success is defined as the achievement of desired job-related outcomes at every point in an individual’s work experiences across time [8]. Employees with successful careers have a greater feeling of welfare and job satisfaction and a lower level of intent to quit; hence, finding the determinants of career success is essential for individuals’ work and professional life [9,10]. Therefore, it can be stated that leadership can help to attain career success in a competitive working environment [3]. There are many different types of leadership style in the literature, but among them, the agile leadership is considered one of the most effective in enhancing the career success of employees because of its flexibility, competency results, and change-oriented approach. Agile leadership is responsive to emerging challenges or opportunities, and it works in quick development cycles of adaptation, learning, and improvement [11]. Agile leadership is a leadership approach that adapts to innovation processes in organizations and allows organizations to survive in competitive environments full of uncertainties [12]. Managers should make an effort to keep flexible workers since they are extremely effective at improving organizational operations and contribute significantly to the organizational success that is critical to enhanced career success [13]. 

Job embeddedness is a management concept developed from the embeddedness theory of Granovetter in 1985 [14], which describes how deeply people are embedded in their job, place, or company [15]. It pertains to the causes of employees remaining in their jobs and persisting in their current careers. Regarding their careers, employees become more productive when they are emotionally involved in the job, company, colleagues teams, and even challenges at work are present. Therefore, they can quickly adapt to changes, both in their organizations and environment. An agile leader encourages followers to find purpose in their jobs and adapt accordingly to predict their future possibilities; however, this effect is related to the employees’ level of job embeddedness. As researchers have stated, career flexibility has a significant social impact on job embeddedness and supports the cognitive and affective aspects of career success [16,17]. As the above studies imply, job embeddedness has a relationship with agile leadership and career success. Taking in account these considerations, this study aimed to see the mediating role of embeddedness in the relationship between agile leadership and perceived career success. 

Thus, we performed a comprehensive analysis of embeddedness in relation to agile leadership and perceived career success. The rest of the paper is organized as follows. The second section reviews the findings of the recent literature in terms of embeddedness, agile leadership, and perceived career success. The third part of the paper presents the variables, data, and methods applied to verify the research hypotheses. The fourth section describes the results of the empirical analysis, while the final discussions and conclusions are discussed in the final parts of the paper.

## 2. Literature Review

As a result of today’s dynamic, difficult, and uncertain business environments, leadership talents are subject to continual depreciation and replacement. To be effective, leaders must be adaptable and flexible enough to adjust their actions and behaviours as circumstances change. Among the most important elements for success in managers and directors, there is their desire and ability to learn from experience paired with changes in the environment [18] and then use that learning to perform well in new situations for career success and job embeddedness. In general, many styles of leadership exist at the organizational level to ensure the organization’s survival. In terms of change velocity, agile leadership is undoubtedly essential for organizational sustainability. Furthermore, in today’s competitive businesses, employee participation and job embeddedness are critical in boosting organizational competitiveness and productivity which are closely related to career success. 

### 2.1. The Link between Agile Leadership and Career Success 

Agile leadership is adaptable to specific issues or opportunities, and it particularly operates in the context of adaptation, learning, and development [11]. Agile leadership is a leadership strategy that adapts to employee empowerment activities and builds organizations’ ability to thrive in unpredictable working scenarios [12]. Agile leaders focus on working as a team rather than individual authority. Since teamwork has been widely explored in management science, it is considered to be very important for career and life satisfaction and career success, making it a significant predictor of the selected career outcome measures [19]. Agile management is most often applied in teams, where personal and social qualities are important to career success. Thus, agile leadership may be defined as a collection of strategies that impact career success in terms of reaching desired outcomes. In order to promote their team vision and company goals, constant group motivation around career success based on feelings is required. This will serve as a guidance and inspiration to their employees to promote their career success.

Most agile leaders are doing an excellent job in their various organizations [20]. There are some other studies on agile leadership available in the literature linking different variables; however, previous studies have overlooked the discussion of the role of agile leadership in career success. In this way, agile leadership is expected to increase the followers’ perceived career success. Thus, we propose:

**Hypothesis** **1** **(H1):**  *There is a significant, positive relationship between agile leadership and career success.*

### 2.2. The Link between Agile Leadership and Job Embeddedness

Most medical and healthcare organizations with diversified professional personnel are managed through hierarchical vertical structures, typically conducted and controlled by administrators. This management strategy, however, restricts the healthcare market and effective responses to a quickly changing healthcare workplace [21]. In contrast to supervisory leadership practices directed towards the maintenance of order and control in an organization, agile leadership improves organizational effectiveness through employees’ efforts, with respect to their capabilities, and performance-motivated volunteer efforts [22]. Agile leadership positively affects individual and team performance, along with job satisfaction [23].

Aspects of being an agile leader, on the other hand, shape how individuals, teams, and organizations view, assess, and interpret work in a dynamic environment, and hence impact their behaviour [23]. Greater agility in an organization has been found to correlate substantially with improved performance, empowerment, competence development, customer orientation, and work satisfaction [24], which are all closely related with job embeddedness. Furthermore, agility and agile leadership in the management of nurses may be directly linked to turnover intention with respect to job embeddedness [25]. Job embeddedness represents the degree to which employees desire to remain in their job and organization, whereas turnover intention is used to discover the factors and processes that prevent job turnover [26]. Job embeddedness has a moderating influence on nurses’ job satisfaction, job stress, and desire to leave [27,28]. Agile leadership and job embeddedness are inextricably linked in the interdependent connection between managers and employees. Therefore, we assume that agile leadership fosters job embeddedness in healthcare organizations and we propose:

**Hypothesis** **2** **(H2):**  *There is a significant, positive relationship between agile leadership and job embeddedness.*

### 2.3. The Link Job Embeddedness and Career Success 

Employees who are well versed in their working environment are able to attain a work–family balance and are usually more pleased with their careers [29,30]. Job embeddedness refers to the pressures that keep one from quitting a job, and these forces get stronger as one’s career advances, making it even more difficult to leave. A high degree of job embeddedness indicates that an employee may form stronger social links with others and live in society with others. Employees begin to feel that the degree of sacrifice required to quit their current work or company is exorbitant. When an employee is thoroughly rooted in both the work and the company, it also indicates that career advancement is considerably more satisfying, and the perceived degree of career success is high. Employees who have less job embeddedness are more likely to engage in job search activities, and they may also have higher degrees of turnover intentions [31]. Employees assess the costs and advantages of job embeddedness, and when the sacrifice of leaving outweighs the benefits of staying in their present position, they elect to stay committed to their professions. They want to keep and protect valuable resources, such as career success and rewards, rather than lose them [4]. These studies show that there is a relationship between job embeddedness and career success. It is therefore hypothesized that job embeddedness significantly contributes to career success:

**Hypothesis** **3** **(H3):**  *There is a significant, positive relationship between job embeddedness and career success.*

### 2.4. The Mediating Role of Job Embeddedness

Several studies have investigated the role of job embeddedness as a moderating factor. Job embeddedness, for example, was found by Lyu and Zhu [32] to moderate the effect of workplace ostracism on emotional commitment and the desire to quit. It is found that job embeddedness is a mediator of the relationship between work–life conflict and turnover intention [33]. In addition, the job embeddedness has a mediating role in the relationships between work–family conflict and voluntary turnover [34,35], due to its moderating influence on family and colleague support for creative performance. Job embeddedness has a mediating role between transformational leadership and career success [36], and between human resource management strategies and employee job performance [37]. 

Therefore, agile leadership is expected to enhance perceptions of career success through job embeddedness. Job embeddedness is positively related to career success [38]. Employees with higher levels of job embeddedness may directly increase their career success and agile leadership may play a key role to enhance job embeddedness and career success. As a result, it is anticipated that job embeddedness can act as a mediation between agile leadership and perceived career success.

**Hypothesis** **4** **(H4):**  *Job embeddedness has a mediating role between agile leadership and team career success.*

**Figure 1:**
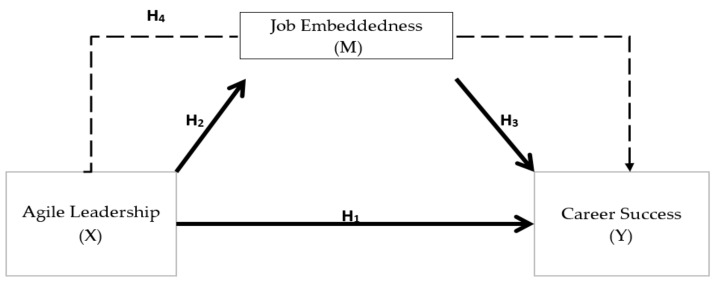
Research model.

Based on the hypotheses above, Figure 1 presents the proposed model of the current study.

## 3. Data and Methodology

### 3.1. Data Collection and Procedures

The present study used a quantitative approach to test the recommended hypotheses. The data were collected using three scales from healthcare organizations. A total of 581 employees in healthcare organizations in Turkey from April to August 2021 participated in the study. All respondents participated in the study voluntarily, and were informed about this. The return of the completed questionnaire was considered as the informed consent. There was a total of 600 target respondents, which is a very good sample, out of which 590 questionnaires were completed and returned, of which 9 questionnaires were found as unusable and discarded. The data were analysed with a sample size of 581 responses; hence, the response rate was 96.8%. The sample size was selected based on Comrey and Lee’s [39] inferential statistics model, where a sample size of 500 is considered to be very good. According to our data, 74.7% of respondents were from public healthcare organizations and 25.3% were from private healthcare organizations. In terms of gender, 34.4% were males and 65.6% were females. In terms of marital status, 70.9% were married while 29.1% were single. As for the companies’ operational areas, almost a quarter of respondents (24.4%) operated in health institutions and 75.6% worked in hospitals. Regarding their education, 19.6% graduated from high school, 65.9% had bachelor’s degree, while 14.5% held a master’s or PhD degree.

### 3.2. Measures

The questionnaire presents four sections. The first section focuses on the sample characteristics of the participants, such as gender, marital status, education level, etc. The second section targets agile leadership as developed by Akkaya et al. [40]. It has six dimensions consisting of 32 items. The third section is about perceived career success measured with a scale of 11 items and developed by Li et al. [41]. The last section relies on participants’ job embeddedness, measured using 7 items developed by Crossley et al. [42]. To measure the items of corresponding variables, a standardized five-point Likert scale was used to organize the scale ranging from 1 (strongly disagree) to 5 (strongly agree).

### 3.3. Analytical Method

This study was designed to utilize a quantitative method approach in order to determine the mediation effect of job embeddedness on the relationship between agile leadership and perceived career success. Our structural equation modelling procedure used AMOS for the examination of causal relationships among variables. 

Structural equation models (SEMs) represent multiple equation regression models where the response variable in one regression equation can be an explanatory variable in another equation [43]. SEM was introduced by Joreskig [44] in the 1970s, including the measurement model and the structural equation model. 

The measurement model indicates how latent variables or hypothetical constructs are explained by the observed variables [45]. The structural equation model reflects the causal relationships among the latent variables, describing the casual effects, and assigning the explained and unexplained variance [46,47].

According to Bollen [48] and Muthen [49], the econometric description of SEM can be reflected by the following two sets of equations:

(1) $$\Gamma y_{i}^{*}+Bx_{i}+E_{i}=0$$

(2) $$y_{i}=h\left(y_{i}^{*},w_{i}\right)+\zeta_{i},\;$$

where the first set of equations represents the latent variable model or the structural model and the second set of equations is the measurement model, *i* denotes the individual, $y_{i}^{*}$ is a (*m* × 1) vector of capability dimensions, ***y****_i_* is a (*p* × 1) vector of functions or indicators, and ***x****_i_* (*q* × 1) and ***w****_i_* (*s* × 1) are vectors of exogenous variables [50].

In this study agile leadership is the exogenous latent variable, while the endogenous latent variable is represented by career success, and job embeddedness is the mediating latent variable. The research model is presented in Figure 1, and the hypotheses are listed below:

**H1:**  *There is a positive relationship between agile leadership and career success in healthcare organizations in Turkey at (p < 0.05) level.*

**H2:**  *There is a positive relationship between agile leadership and job embeddedness in healthcare organizations in Turkey at (p < 0.05) level.*

**H3:**  *There is a positive relationship between job embeddedness and career success healthcare in organizations in Turkey at (p < 0.05) level.*

**H4:**  *Job embeddedness has a mediating role between agile leadership and career success in healthcare organizations in Turkey at (p < 0.05) level.*

## 4. Empirical Results

Before evaluating the data, several statistical parameters were checked to ensure that the data were distributed normally. The data distribution should be normal for parametric tests, such as the ANOVA, regression, and structural equation model tests [51]. 

The skewness and kurtosis values were between +1 and −1, as seen in Table 1. As a result, we employed SEM as one of the parametric analyses. 

Model revisions were carried out based on assessments of factor loadings, standardized residuals (SRs), and modification indices (MIs), while maintaining the concentricity of the measurement model within the theoretical framework. Items with factor loadings of <0.3 were considered for removal. The factor loadings of each item should exceed 0.30, representing an acceptable fit [52]. To test the reliability of the scales, Cronbach alpha (α) was calculated. As seen in Table 1, the Cronbach alpha (α) value was bigger than 0.70 for all scales, indicating the reliability of the scales [53]. Furthermore, the reliability of the six dimensions of agile leadership was presented through the alpha (α) values as follows: teamwork-oriented was 0.73; result-oriented was 0.79; competency was 0.73; flexibility was 0.72; quickness was 0.70; and change-oriented was 0.72.

Firstly, we used correlation analysis to determine the link between variables in the study model. The results are shown in Table 2. Table 2 shows that there is a medium correlation between variables.

**Figure 2:**
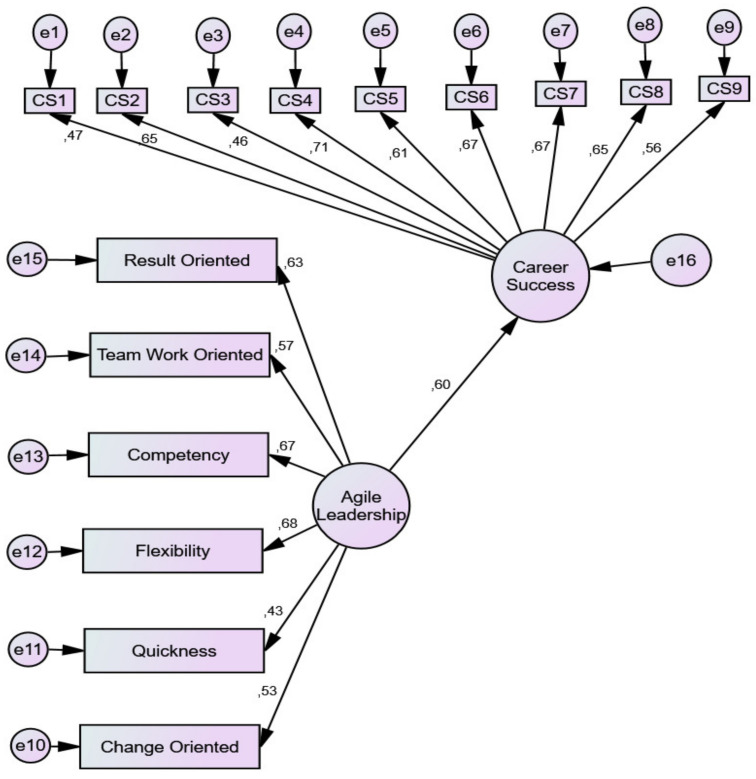
Direct effect of agile leadership on career success.

According to Baron and Kenny [54], the mediator functions to mediate any correlated relationship between the endogenous and exogenous latent. Figure 2 illustrates the modelling path of the direct effect of agile leadership on career success, which must be significantly direct to test the mediation effect of job embeddedness. Figure 2 shows that the direct effect of agile leadership on career success is significant. In other words, agile leadership has an impact on career success (*p* < 0.05). Therefore, Hypothesis H1 can be accepted. 

When the mediating variable of job embeddedness was introduced into the model, the value of the beta coefficient for career success was predicted to decrease, implying that the direct influence of agile leadership on career success was diminished (Table 3).

**Figure 3:**
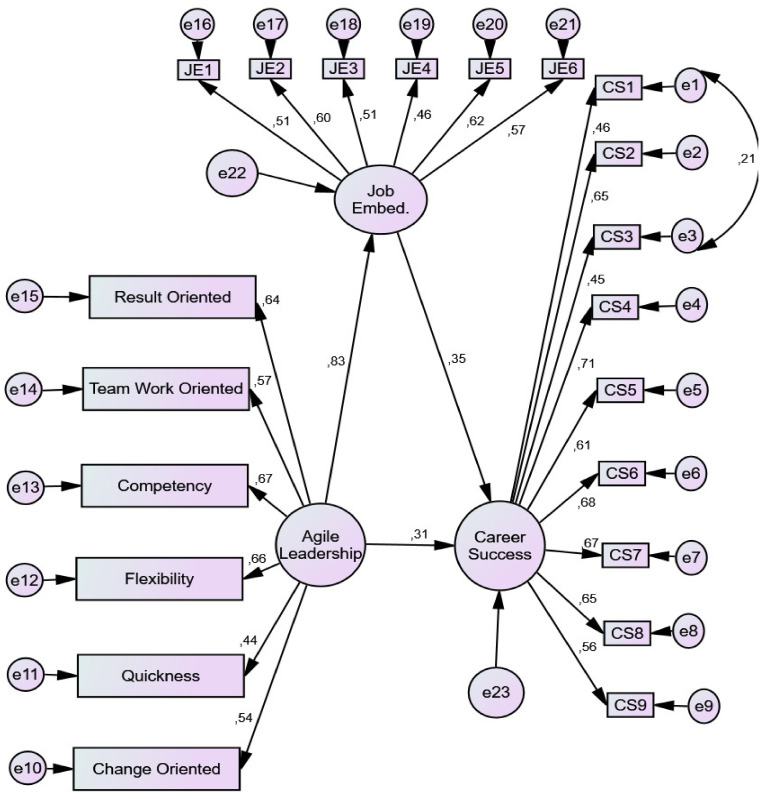
Regression coefficient between all constructs.

Figure 3 depicts the research model using job embeddedness as a mediator.

The value for CMIN (χ²) was 2.43; *p* was 0.000; RMSEA was 0.050; RMR was 0.57; CFI was 0.929; IFI was 0.920; and GFI was 0.923. In Table 4, the value of the beta coefficient linking agile leadership to career success reduced from 0.60 to 0.31. In this case, agile leadership was significant in terms of direct effect on career success and indirectly significant in terms of effect on career success through a mediator variable, namely job embeddedness. 

As seen in Table 4, there was a positive relationship between agile leadership and interpersonal job embeddedness (*p* < 0.05). Therefore, H_2_ can be accepted. Moreover, it can be assumed that job embeddedness can influence career success either directly or indirectly (*p* < 0.05). Therefore, H_3_ can also be accepted. The direct effect of agile leadership on career success was still partially significant even after job embeddedness enters the model and even though the beta coefficient for agile leadership was reduced from 0.60 to 0.31. Thus, we concluded that there was partial mediation effect of job embeddedness on the relationship between agile leadership and career success. Therefore, H_4_ can also be accepted.

## 5. Conclusions

This study examined the mediation effect of job embeddedness on the relationship between agile leadership and career success. The total mediation effect of job embeddedness on the relationship between agile leadership and career success was confirmed. This study has four important implications and findings for management theory and practice. Firstly, agile leadership can promote the perception of career success among healthcare employees. This is in line with Dai et al. [55] who found that agile leadership positively impacted career success, and is also line with Purdy [56] who found that leadership positively affected career success. These studies supported Hypothesis 1.

Secondly, the characteristics of an agile leader empower employees to accept changes in the environment and become more flexible in the complex, unpredictable, and dynamic environment in their organization. A high-quality leader’s behaviours supply employees with resources linked to job embeddedness. This finding, in other words, supports Hypothesis 2, in agreement with research conducted by Dechawatanapaisal [28], which found that leaders with high-quality behaviours could have an impact on job embeddedness. Thirdly, this study discovered that embedded employees believe their careers are progressing in the proper direction, giving them contentment. Stumpf [38] and Al-Ghazali [4] supported Hypothesis 3. Stumpf [38] discovered that workers with higher job embeddedness were less inclined to change jobs or organizations and were more successful in their career development. Al-Ghazali [4] found that job embeddedness significantly influenced the career success of nurses and Dedeoğlu et al. [57] found that there was a positive relationship between work embeddedness and optimism. 

Finally, as hypothesized in Hypothesis 4, employee job embeddedness plays a mediating role in the connection between agile leadership and career success. According to this partial mediation, the influence of agile leadership on career success is partially mediated through job embeddedness. This means that employees with a positive perception of the organization helps them to make sense of their work and creates positive relationships between leaders and employees that embed them into their organization, therefore making it more difficult for them to leave. There are some studies which support this hypothesis. Kim and Kim [58] found that job embeddedness had a mediating effect between self-efficacy and self-leadership in nursing. Karanika-Murray et al. [59] supported this finding by stating that a strong link between the employee and their company is a crucial requirement for their bond with their work, and it is highly connected to attitudinal and behavioural outcomes. Moreover, Afsar and Badir [60] and Al-Ghazali [4] found that job embeddedness has a mediating role in the relationship between employees’ perceived organizational support and organizational employee behaviour and leadership. An overview of the results and relevant aspects derived from the results is presented in Table 5. 

As leaders play an important role in motivating and pushing subordinates to reach their full potential in any company [61,62,63,64,65,66,67,68,69,70,71,72,73], more research is needed to understand the impact of agile leadership on employees’ perceived career success. Thus, this study addressed the research by examining the relationship between agile leadership, job embeddedness, and perceived career success. Furthermore, it also examined the mediating effect of career job embeddedness to provide a better understanding of the mechanism through which agile leaders promote career success.

### Limitations and Future Research

Despite the uniqueness of the research subject, the authors recognize the study’s limitations. These constraints are related to the quantitative examination of respondents’ impressions of the topics under consideration. Furthermore, restricting the research sample to just healthcare companies limits the scope of the findings. Further research directions should include an examination of the relationship between leadership and career success in various countries and departments, an in-depth examination of respondents’ perceptions using qualitative tools, and an examination of specific instruments promoting the formation of job embeddedness in agile leaders.

## Figures and Tables

**Table 1:** Descriptive statistics.

	N	Mean	Std. Deviation	Skewness		Kurtosis		
	Statistic	Statistic	Statistic	Statistic	Std. Error	Statistic	Std. Error	(α)
Agile leader	581	3.814	0.702	−0.304	0.101	−0.231	0.202	0.74
Career success	581	3.662	0.597	−0.298	0.101	−0.303	0.202	0.84
Job embeddedness	581	3.786	0.654	−0.266	0.101	−0.454	0.202	0.72
Valid N (listwise)	581							

**Table 2:** Correlations.

		Agile Leader	Career Success	Job Embeddedness
Agile leader	Pearson Correlation	1		
	Sig. (2-tailed)			
	N	581		
Career success	Pearson Correlation	0.599 **	1	
	Sig. (2-tailed)	0.000		
	N	581	269	
Job embeddedness	Pearson Correlation	0.481 **	0.482 **	1
	Sig. (2-tailed)	0.000	0.000	
	N	581	581	581

**. Correlation is significant at the 0.01 level (2-tailed).

**Table 3:** Direct effect of agile leadership on career success.

			Estimate	S.E.	C.R.	P	CMIN/DF	CFI	GFI	IFI	RMSEA	RM
**H1:** Career_Success	⤎	Agile Leadership	0.579	0.072	8.031	***	2.869	929	943	930	0.057	0.057

*** The model is significant at the 0.001 level.

**Table 4:** Multiple regression weight and hypotheses testing.

Direct Paths			Estimate	S.E.	C.R.	P
**H2:** Job_Embed.	⤎	Agile_Leadership	0.892	0.092	9.368	0.000
**H3:** Career_Success	⤎	Job_Embed.	0.312	0.114	2.741	0.006
**H4:** Career_Success	⤎	Agile_Leadership	0.292	0.114	2.566	0.010

**Table 5:** Summary of the reseach.

Results	Relevant Aspects	Researches Supported
A positive relationship between agile leadership and career success	Agile leadership can promote the perception of career success among healthcare employees	Dai et al. [55] Purdy [56]
A positive relationship between agile leadership and job embeddedness	The characteristics of an agile leader empower employees to accept changes in environment and become more flexible. A high-quality leader’s behaviours supply employees with resources linked to job embeddedness	Dechawatanapaisal [28]
A positive relationship between job embeddedness and career success	Embedded employees believe their careers are progressing in the proper direction and were more successful in their career development.	Stumpf [38 ] Al-Ghazali [4] Stumpf [38] Dedeoğlu et al. [57]
A mediating role job embeddedness between agile leadership and career success	A positive perception of employees with the organization helps them to make a better sense and creates the collection of positive relationship between leaders and employees.	Kim and Kim [58] Karanika-Murray et al. [59] Afsar and Badir [60] Al-Ghazali [4]

## Data Availability

Not applicable.
